# Reply to Červenák et al., “Primary Scientific Literature Represents an Essential Source of Telomeric Repeat Sequences”

**DOI:** 10.1128/spectrum.01533-23

**Published:** 2023-06-13

**Authors:** Qing Sun, Hao Wang, Shiheng Tao, Xuguang Xi

**Affiliations:** a College of Life Sciences, Northwest A&F University, Yangling, Shaanxi, China; b Bioinformatics Center, Northwest A&F University, Yangling, Shaanxi, China; c State Key Laboratory of Crop Stress Biology in Arid Areas, Northwest A&F University, Yangling, Shaanxi, China; d Université Paris-Saclay, ENS Paris-Saclay, CNRS, LBPA, Gif-sur-Yvette, France; The Ohio State University

## REPLY

We appreciate the interest and comments from Červenák et al. on the *Microbiology Spectrum* article “Large-scale detection of telomeric motif sequences in genomic data using TelFinder” (1). Červenák et al. mainly stressed the importance of primary literature resources in discussing the current understanding of telomeric repeat sequences. To clarify these concerns adequately, we address each query separately.

The first question posed by Červenák et al. concerns telomeric repeat sequences being identified in only a small number of fungal genomes in the NCBI database. One of the factors considered in the TelFinder algorithm is SuppChr, which is the number of chromosomes with the telomeric repeat candidates. In genomes with a lower degree of assembly, the scaffolds or contigs can be considered to be chromosomes in the detection step of TelFinder. Since there are also some repeated sequences within the genome, the confidence in the given possible telomeric motif sequence will undoubtedly be affected. Therefore, it is recommended to use genomes whose assembly level is higher than the chromosome level for detection with TelFinder. Although there are 4,148 representative fungal genomes in the NCBI genome database, only approximately 3% of the genomes were assembled at a level above the chromosome level. Therefore, we detected telomeric repeats in these fungi.

The second question concerns the genus names of species being erroneous in Fig. 3 (i.e., *Saccharomycopsis malanga* and Saccharomycopsis fibuligera belong to the genus *Saccharomycopsis*, and Yarrowia lipolytica belongs to the genus *Yarrowia*). We have performed a thorough examination of the relevant text, figures, and tables to ensure that all the species assignments are correct. We thank Červenák et al. for their careful review, and we will correct these mistakes.

The third question concerns telomeric motif sequences being mislabeled in Fig. 2. On the basis of a careful review of the detection result, TGTACGGATGTCTAACTTCTTGG is the telomeric motif sequence in Candida albicans detected by TelFinder, consistent with that in the literature (2), but it was mislabeled in Fig. 2 and Table S1. Similarly, the telomeric motif sequence of Kluyveromyces lactis is TTTGATTAGGTATGTGGTGTACGGA in the detection result from TelFinder, which is consistent with that in the report (2), but it was mislabeled in Fig. 2 and Table S1. We will correct the telomeric motif sequences of C. albicans and K. lactis in the corresponding figure and table. In addition, the telomeric motif sequences in Candida tropicalis can differ among strains. As reported by McEachern et al., A[C/A]GGATGTCACGATCATTGGTGT is the telomeric motif sequence in strains B-4414 and 1739-82 of C. tropicalis, while B-4438 and B-4439 carry only AAGGATGTCACGATCATTGGTGT telomeres (2). In the detection result from TelFinder, GTGTAAGGATGTCACGATCATTG was the most likely telomeric repeat candidate, consistent with that in strains B-4443, B-4438, and B-4439. Therefore, we think that the detection result is consistent with the report. We also checked all sequences in Table S1 and confirmed that they were not labeled incorrectly and were consistent with those in the responding literature.

The fourth problem concerns the timeliness and accuracy of the Telomerase Database. Due to a lack of recent updates, the telomeric motif sequences recorded in the database are incomplete. However, there is no other detailed database of telomere sequences at present. Moreover, the information from the Telomerase Database was used to verify the performance of TelFinder. TelFinder successfully detected telomeric repeat sequences in these species, and some false records were also recognized. These issues do not affect the performance of TelFinder. We fully agree that the primary literature resources are helpful when discussing the current understanding of telomere repeat sequences. Accordingly, some reported telomeric motif sequences were mistakenly declared to be novel discoveries, which will be corrected to avoid misleading readers.

The focus of our paper is the development of TelFinder to detect telomeric motif sequences quickly and efficiently. The inadequacy of the Telomerase Database does not affect the tool’s efficiency or any conclusions of the paper.

## References

[1] Sun Q, Wang H, Tao S, Xi X. 2023. Large-scale detection of telomeric motif sequences in genomic data using TelFinder. Microbiol Spectr 11:e03928-22. doi:10.1128/spectrum.03928-22. 36847562 PMC10100673

[2] McEachern MJ, Blackburn EH. 1994. A conserved sequence motif within the exceptionally diverse telomeric sequences of budding yeasts. Proc Natl Acad Sci USA 91:3453–3457. doi:10.1073/pnas.91.8.3453. 8159768 PMC43595

